# Important Risk Factors in Patients with Nonvalvular Atrial Fibrillation Taking Dabigatran Using Integrated Machine Learning Scheme—A Post Hoc Analysis

**DOI:** 10.3390/jpm12050756

**Published:** 2022-05-06

**Authors:** Yung-Chuan Huang, Yu-Chen Cheng, Mao-Jhen Jhou, Mingchih Chen, Chi-Jie Lu

**Affiliations:** 1 Graduate Institute of Business Administration, Fu Jen Catholic University, New Taipei City 242062, Taiwan; richardych1111@gmail.com (Y.-C.H.); aaa73160@gmail.com (M.-J.J.); 081438@mail.fju.edu.tw (M.C.); 2 Department of Neurology, Fu Jen Catholic University Hospital, Fu Jen Catholic University, New Taipei City 24352, Taiwan; vattmo@gmail.com; 3 Artificial Intelligence Development Center, Fu Jen Catholic University, New Taipei City 242062, Taiwan; 4 Department of Information Management, Fu Jen Catholic University, New Taipei City 242062, Taiwan

**Keywords:** arrhythmia, cardioembolic stroke, non-vitamin K antagonist oral anticoagulants, dabigatran, machine learning

## Abstract

Our study aims to develop an effective integrated machine learning (ML) scheme to predict vascular events and bleeding in patients with nonvalvular atrial fibrillation taking dabigatran and identify important risk factors. This study is a post-hoc analysis from the Randomized Evaluation of Long-Term Anticoagulant Therapy trial database. One traditional prediction method, logistic regression (LGR), and four ML techniques—naive Bayes, random forest (RF), classification and regression tree, and extreme gradient boosting (XGBoost)—were combined to construct our scheme. Area under the receiver operating characteristic curve (AUC) of RF (0.780) and XGBoost (0.717) was higher than that of LGR (0.674) in predicting vascular events. In predicting bleeding, AUC of RF (0.684) and XGBoost (0.618) showed higher values than those generated by LGR (0.605). Our integrated ML feature selection scheme based on the two convincing prediction techniques identified age, history of congestive heart failure and myocardial infarction, smoking, kidney function, and body mass index as major variables of vascular events; age, kidney function, smoking, bleeding history, concomitant use of specific drugs, and dabigatran dosage as major variables of bleeding. ML is an effective data analysis algorithm for solving complex medical data. Our results may provide preliminary direction for precision medicine.

## 1. Introduction

Stroke is the leading cause of death and disability worldwide [1]. Cardioembolic stroke is a primary subtype, and nonvalvular atrial fibrillation (NVAF) is one of the most common risk factors for cardioembolic stroke, with a global prevalence of 1–2% [2]. In recent decades, this event has been treated by shifting from the traditional vitamin K antagonist warfarin to nonvitamin K antagonists (NOACs) [3,4,5]. Because of the significant increase in the clinical demands for NOACs, off-label use, especially the dosage selection regimen, has become an important issue in recent years. In real-world studies, off-label low-dose NOACs were prescribed to approximately 9–31% of patients with NVAF [6,7]. Adverse effects including a higher risk of ischemic stroke and systemic embolism have been observed in these patients [8,9].

Dabigatran etexilate, the only direct thrombin inhibitor, is an NOAC with two approved doses based on the Randomized Evaluation of Long-Term Anticoagulant Therapy (RE-LY) trial [10]. In this trial, low-dose dabigatran (110 mg twice daily) had similar vascular prevention effects as those of warfarin with lower rates of major hemorrhage. High-dose dabigatran (150 mg twice daily) was associated with lower rates of vascular events but similar rates of major hemorrhage. The dosage adjustment plan of dabigatran was based on previous studies and expert opinions (European label) [11], which suggested that clinicians could decrease the dosage of dabigatran among patients aged >80 years, those aged 75–80 years with a high risk of bleeding, or those with concomitant use of verapamil. Physicians must balance the risk of recurrent stroke and bleeding tendency in clinical practice. Currently, the congestive heart failure, hypertension, age ≥ 75 years [doubled], diabetes mellitus, prior stroke, transient ischemic attack or thromboembolism [doubled], vascular disease, age 65–74 years, and sex category (CHA_2_DS_2_-VASc) [12] and hypertension, abnormal renal/liver function, stroke, bleeding history or predisposition, labile international normalized ratio, elderly [age ≥ 65 years], drugs/alcohol concomitantly (HAS-BLED) [13] scores are used to calculate the risk of recurrent ischemic stroke and assess bleeding risk, respectively. However, these tools share the same grading factors: old age, hypertension, and stroke history. This may lead to a clinical dilemma, i.e., one patient could score high in both scoring systems. Although the CHA_2_DS_2_-VASc score has been widely used for years with convenience and reliability [14,15], insufficient prediction performance (C statistic of 0.679) has remained a concern [16]. Machine learning (ML) methods have been recently used as well-constructed analytical, classification and prediction tools for medical problems [17,18,19,20,21,22]. Their advantage and performance in demonstrating complex relationships between risk factors and outcomes and analyzing important information hidden in the vast amount of medical data have made them an emerging research topic. Kamel et al. [23] and Chun et al. [24] have confirmed the feasibility of predicting vascular events. Unlike prediction models that use only one ML technique that might be insufficient to provide complete, adequate and stable feature selection results, our study developed an integrated ML feature selection scheme with the benefits of stable and balanced performance. Our method may reveal important variables influencing the efficacy and safety of dabigatran to provide a precision medical suggestion regarding dose selection and risk control for patients with different characteristics.

## 2. Materials and Methods

### 2.1. Study Population

This study is a post-hoc analysis based on RE-LY trial dataset. This study was reviewed and approved by the Research Ethics Review Committee of the Fu Jen Catholic University Hospital. The requirement for informed consent was waived, since the data contain only de-identified information.

In the RE-LY trial, >18,000 patients with newly diagnosed arrhythmia and indications of secondary prevention with an anticoagulant were randomized to receive dabigatran 110 or 150 mg twice daily or an adjusted dose of warfarin with a median follow-up period of approximately 2 years. Exclusion criteria included a history of severe heart valve disorders, a recent stroke, and renal insufficiency. The primary outcome was stroke or systemic embolism and the primary safety outcome was major hemorrhage. The definitions and results of other secondary outcomes have been described in detail and published. We collected the data of patients taking dabigatran with complete follow-up in the RE-LY trial for the present analysis.

### 2.2. Proposed Integrated Machine Learning Scheme

**Figure 1:**
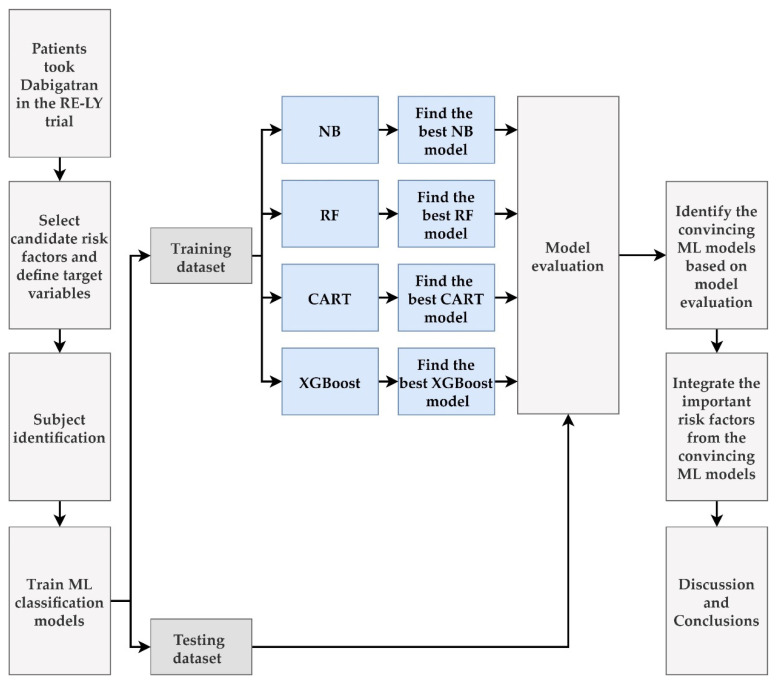
Flow chart of the proposed integrated ML feature selection scheme.

We proposed an integrated ML feature selection scheme for predicting vascular events and bleeding in patients with NVAF taking dabigatran and for identifying important risk factors. Figure 1 shows the process of establishing the proposed scheme.

First step: Identify risk factors as predictor variables and define target variables. For risk factors we referred to the recommendations in the guidelines of the American Heart Association and the European Society of Cardiology [11,25], which included sex; age; body mass index (BMI); body weight; ethnicity; kidney function abnormality; concomitant use of specific drugs; history of hypertension, stroke, previous bleeding, myocardial infarction (MI), diabetes mellitus (DM), congestive heart failure (CHF), or systemic embolism; smoking; and liver function abnormality. Boundaries of subgroups in most variables followed the definition of CHA_2_DS_2_-VASc and HAS-BLED scores. BMI was classified according to the definition of the World Health Organization [26]. Moderate and severe kidney function abnormality was labeled according to the United States Food and Drug Administration (USFDA) [27].

For analyzing the influence of these factors on efficacy and safety, we selected two target variables including vascular events (P1: stroke, MI, systemic embolism, and vascular death) and major bleeding (P2: major bleeding defined as blood loss with a decrease in hemoglobin level of ≥2 g/dL (1.2 mmol/L), transfusion of ≥2 packed red blood cells, or symptomatic bleeding in a critical area or organ). Our presumed important variables and prognostic outcomes were individually categorized according to the definition shown in Table 1.

Subjects were identified according to participants’ characteristics and laboratory data collected during their enrolment in the RE-LY trial. Only patients with available complete information were included in our analysis. Two independent investigators confirmed prognostic outcomes according to the criteria mentioned in the trial.

The study protocol included one traditional prediction method, logistic regression (LGR), and four ML techniques, viz., naive Bayes (NB), random forest (RF), classification and regression tree (CART), and extreme gradient boosting (XGBoost). NB is a popular ML model used for classification tasks. This algorithm can sort objects according to specific characteristics and variables based on the Bayes theorem. It calculates the probability of hypotheses on presumed groups [28]. RF is an ensemble learning method developed by constructing several decision trees. It collects numerous random samples of variables as the training dataset to alleviate the overfitting feature of decision trees. Each tree in the RF outputs its prediction result, and the class with the most votes sums up the best performance model [29]. CART is a classification ML algorithm that constructs a decision tree based on Gini’s impurity index. The decision tree structure comprises root, internal, and leaf nodes, which may represent training data and decision-making points. The CART prediction model is constructed by picking variables and evaluating split ends until an appropriate tree is produced [30]. XGBoost is an optimized distributed gradient boosting system that implements ML algorithms based on the gradient boosting framework. It uses the regularization term to control model complexity and simultaneously uses first- and second-order derivatives to perform a second-order Taylor expansion of the loss function [31]. These ML methods, which share characteristics of interpretable tools for prediction and classification with good performance in vast unprocessed data, have been widely applied in solving cerebrovascular and cardiovascular disease problems [32,33,34,35]. Meanwhile, the logistic regression, which is a widely accepted analytic method in medical research, was defined as the benchmark in our study.

Second step: Train NB, RF, CART, and XGBoost models and evaluate their predictive performance. The models are trained using two combinations of predictor and target variables. One combination involves using 18 variables (V1–V18) as predictors and vascular events (P1) as the target variable; the other combination involves using V1–V18 as predictors but bleeding (P2) as the target variable. In training these models, the data of recruited patients were randomly separated into 90% training and 10% testing datasets according to the 10-fold cross-validation (CV) method. Our scheme applied the 10-fold nested CV method for enhancing stability to estimate the best performance of each model [36]. This process consisted of 10-fold inner CV for tuning and then determining the best hyperparameter set of each method for model selection and 10-fold outer CV for evaluating the predictive performance of the best model of each method for model evaluation.

These models’ efficacy were evaluated based on their mean and standard deviation of accuracy, sensitivity, specificity, and area under the receiver operating characteristic (ROC) curve (AUC) [37]. Sensitivity is the proportion of true positives tests of all patients with predicted events. Specificity is the proportion of true negative tests out of all patients who have not predicted events. Accuracy is the proportion of correct predictions (both true positives and true negatives tests) among the total number of patients examined. ROC curve is a graphic performance measurement of a classification model at various classification thresholds. AUC is the Area under the ROC curve, which provides an aggregate performance measure across all possible classification thresholds. The best hyperparameters with leading validation performance based on the AUC value for each model can be chosen to construct tuned NB, RF, CART, and XGBoost best models. The results of the best performance model with AUC values exceeding those of LGR were the cornerstone of our predicting models of vascular events and bleeding.

Third step: Importance ranking of risk factors. The “caret” R package version 6.0-90 [38] was applied for each of the four methods to generate each variable’s importance value. We defined the priority demonstrated in each model ranking 1 as the most critical factor and 18 as the least critical factor. Each model would perform 10 times due to the use of 10-fold outer CV to gain the average ranking of each variable for more confident results. Individual ML methods may produce different importance rankings owing to distinct characteristics. Ensemble machine learning method based on a combination of multiple models’ outputs is widely accepted and has produced good results in recent years [39]. An integrated ML feature selection scheme might assemble the prediction powers of these methods. We summarized the major important variables from the average ranking of each risk factor based on the identified convincing ML models to enhance stability and integrity.

According to the individual priority of the variables presented in the predictive models of vascular events and bleeding, we may establish an instruction concept for patients with NVAF taking dabigatran. In the final stage, we summarized our significant findings and discussed them in light of previous concepts.

All analyses were conducted using R software version 4.1.2 (R core team, Vienna, Austria) and RStudio version 1.1.453 (http://www.R-project.org; accessed on 2 March 2022; https://www.rstudio.com/products/rstudio/; accessed on 2 March 2022). The methods were applied using the R software with the required installed packages: “randomForest” package version 4.6-14 for RF [40]; “rpart” R package version 4.1-15 [41] for CART; and “XGBoost” package version 1.5.0.2 for XGBoost [42]. To estimate the best parameter set for developing effective RF, CART, and XGBoost methods, the “caret” package version 6.0-90 [38] was used for tuning the relevant hyperparameters. NB was implemented using the “klaR” package version 0.6-15 [43] with the default setting of hyperparameters.

## 3. Results

There were 12,091 patients randomized to take dabigatran in the RE-LY trial. After excluding 289 patients with missing data, 11802 patients were enrolled in our study. Subjects’ demographic data are outlined in Table 2. There were 318 (2.69%) patients with vascular events, and 2238 (18.96%) patients had bleeding within the first year of follow-up when taking dabigatran, while others were event-free.

**Figure 2:**
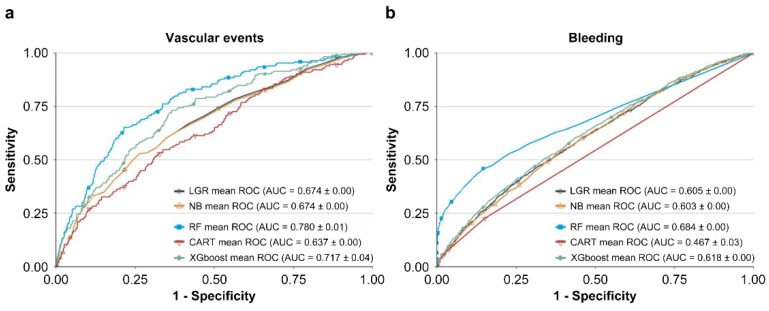
Receiver operating characteristic (ROC) curves of the five methods in predicting (**a**) vascular events and (**b**) bleeding.

Table 3 shows the values of hyperparameters which train best NB, RF, CART, and XGBoost models with leading AUC values. The performances of LGR, NB, RF, CART, and XGBoost methods in predicting vascular events and bleeding are listed in Table 4. The ROC curve of each model is presented in Figure 2. In predicting vascular events, RF (AUC = 0.780) and XGBoost (AUC = 0.717) showed higher AUC values than LGR (AUC = 0.674). In predicting bleeding, RF (0.684) and XGBoost (0.618) showed higher AUC values than LGR (0.605). In contrast, NB and CART showed inferior performance to LGR in predicting vascular events and bleeding. Therefore, we selected RF and XGBoost as the basis of our integrated ML feature selection model.

Table 5 shows the overall importance ranking of each risk factor in predicting vascular events based on RF and XGBoost. The average rankings with 10-fold cross-validation of the two models were demonstrated as “Average Ranking of 10 Times RF” and “Average Ranking of 10 Times XGBoost”. The different methods generated individual importance ranking according to their analyzing rules. For a more comprehensive view, we summarized the findings of the two models equally in our integrated ML feature selection scheme. We obtained the “Average ranking of the 2 Models” with simple averaging the average ranking values from the RF and XGBoost models. To clarify the ranking, we ranked the result from 1 and showed that the “Final ranking in predicting vascular events” was listed using the “Average ranking of the 2 Models” value. Age; history of CHF, MI, DM, and stroke; smoking; kidney function; BMI; ethnicity, and dabigatran dosage were the major predictor variables of vascular events.

Table 6 presents the overall importance ranking of each risk factor in predicting bleeding. By averaging the rank values of RF and XGBoost methods, we concluded that age, kidney function, smoking, bleeding history, concomitant use of specific drugs, dabigatran dosage, BMI, MI and CHF history, and ethnicity were the major predictor variables of bleeding.

## 4. Discussion

To our knowledge, this is the first study attempting to analyze risk factors in patients with NVAF taking dabigatran using integrated ML feature selection methods. RF and XGBoost demonstrated prominent prediction values exceeding those of LGR. We could conclude the ranking of essential risk factors in these patients after averaging the results of these two methods. In order to balance simplicity and practicality against precision, we selected the top nine important variables to discuss according to physicians’ decision. (Table 7).

In most predictive models, an age of >65 years is a standard variable that predicts ischemic stroke and bleeding. As expected, age was the leading predictor of vascular events and bleeding in our study.

Smoking induces atherosclerosis and endothelial dysfunction, simultaneously resulting in more ischemic insults and hemorrhage [44,45,46]. Smoking also contributes to an increased probability of developing arrhythmia via several metabolic factors and underlying diseases [47]. Smoking cessation is a well-documented strategy to prevent vascular disease either with or without arrhythmia. Regarding the medical management of patients with atrial fibrillation, smoking has received insufficient attention. In a consensus, smoking was reported to increase warfarin clearance, influencing the drug effects [48]. There was no similar concern when the anticoagulant was shifted to NOACs. However, in our study, smoking appeared to be a more important variable than other common systemic diseases in patients taking dabigatran.

In the CHA_2_DS_2_-VASc score, ischemic stroke history played a more critical role than MI after adding the two scores when patients ever had a stroke. However, MI was a prevalent risk factor for vascular events rather than stroke in our study. CHF that might result from ischemic heart disease or hypertension complications also has a significant impact on most evaluating tools [49]. Cardiomegaly caused by these underlying diseases leads to left ventricular hypokinesia, the major cause of thrombus formation [50,51,52]. However, endothelial dysfunction and cerebral autoregulation disturbance are also the consequences of CHF [53]. In ML models, we may comprehensively analyze several variables with different interactions; hence, CHF and MI show higher grades in the prediction of vascular events among all underlying diseases.

Kidney dysfunction was infrequently mentioned as a major risk factor for ischemic stroke. Severe kidney impairment (estimated creatinine clearance <30 mL/min/1.73 m^2^) was an exclusion criterion for most NOACs [10,54,55,56]. Delayed drug clearance may increase the possibility of bleeding [57]. In a study on Danish population, kidney function impairment was found to contribute to a high tendency of developing vascular events and bleeding [58]. The levels of inflammatory and procoagulant factors including C-reactive protein, fibrinogen, factor VIIc, and factor VIIIc were high [59]. Furthermore, hemostatic dysfunction including decreased glycoprotein IIb and IIIa levels, reduced von Willebrand factor activity, and altered arachidonic acid metabolism were detected in older individuals with renal insufficiency [60]. These double-sided adverse effects may be due to these physiological alterations, and kidney dysfunction is the end-organ damage result of hypertension and diabetes. Our scheme selects it as a significant representative variable of vascular events and bleeding.

In general, a high BMI may be associated with metabolic syndrome and hypertension [61]. High BMI increases the prevalence of cerebrovascular and heart diseases [62]. However, this trend is controversial in patients with arrhythmia. Meta-analysis and real-world cohort studies have revealed less ischemic stroke and bleeding prevalence in patients with high BMI [63,64]. The all-cause death rate was higher in underweight patients. BMI was critical for predicting vascular events and bleeding in our study.

For a competitive relationship in the CYP3A4 and P-glycoprotein inhibition pathway [65], the recommended dabigatran dose in the European label is 110 mg if a patient is on verapamil. In the United States, the USFDA recommended that clinicians use dabigatran with caution when patients are on long-term use of nonsteroidal anti-inflammatory drugs (NSAIDs), antithrombotic agents, or medicines that may elevate the blood levels and effects of dabigatran, such as dronedarone or ketoconazole [66]. Observational studies conducted in the US and Taiwan have indicated that concomitant use of these drugs enhances bleeding risk in patients taking dabigatran [67,68]. Combining antithrombotic and antiplatelet agents is a well-known therapy limited in certain conditions owing to high bleeding probability [69]. Our results confirm that these drug interactions have an important effect on bleeding risk.

This study attempted to solve the dilemma of dose selection of dabigatran to obtain the maximum benefit of prevention and avoidance of side effects in patients with various physiological conditions and comorbidities. Dabigatran dose was also defined as a variable in our model. Although it had a noticeable influence on both vascular events and bleeding, it was not a major factor in either result. This issue remains a complex problem that our study could not solve completely because of three of the top five risk factors of either vascular event or bleeding being the same (Table 7). Nevertheless, we could identify essential factors to provide good suggestions using this model. First, smoking cessation and maintaining an appropriate body shape are vital for patients prescribed dabigatran. CHF and MI imply a high risk for thrombotic events with secondary prevention with dabigatran, and intensive medical control and prescribing a standard dabigatran dose are essential. In contrast, a previous bleeding history and concomitant use of antithrombotic agents, NSAIDs, and medicines with effects on CYP3A4 have adverse effects on bleeding when we select a low dabigatran dose. However, older age and kidney function impairment have double-sided adverse effects causing more vascular events and bleeding simultaneously. Other methods are indicated to determine the dividing line of each factor if it exists.

## 5. Limitations

Our study has several limitations. First, our findings must be applied with caution in clinical practice considering the inclusion and exclusion criteria of the RE-LY trial. The trial population comprised subjects with relatively low CHA_2_DS_2_-VASc scores (2.1 ± 1.1), and patients with certain comorbidities were excluded. Second, the trial participants were regularly followed up for two years with good compliance that might be infrequent in our outpatients. Specific effects of other systemic diseases might be alleviated. Third, we intended to establish a prediction model for patients with NVAF taking dabigatran; vascular events including stroke, MI, systemic embolism, and vascular death were defined as the primary outcome. Given that we selected only one NOAC instead of an antiplatelet agent or combined therapy, and though an antiplatelet agent could prevent atherosclerosis, this issue might be affected by risk factors including dyslipidemia, lifestyle, and genetics, which were not included in our study. Nevertheless, our study design was suitable for clinical practice when considering the secondary prevention of cardioembolic stroke.

## 6. Conclusions

NOACs could replace warfarin owing to their similar protective effects and better safety quality in real-world studies. Appropriate dose selection and intensive risk factor control are necessary to achieve high-quality secondary prevention. In our research, RF and XGBoost generated higher accuracies and AUC values than LGR in simultaneously predicting vascular events and bleeding even with the disproportionate prevalence. Furthermore, these methods remained relatively stable between their sensitivities and specificities in the imbalanced data. This integrated ML feature selection scheme showed a great opportunity to solve complex medical data. Although further evaluation is indicated, our study might provide a preliminary direction of precision medicine for secondary prevention in patients with arrhythmia.

## Figures and Tables

**Table 1:** Description of predictor and target variables in this study.

	Variables	Description	Units
V1	Sex	**0**: Male; **1**: Female	-
V2	Age	**1**: <65; **2**: ≥65 and <75; **3**: ≥75	years
V3	BMI	**1**: <18.5; **2**: ≥18.5 and <30; **3**: ≥30	kg/m^2^
V4	Body weight	**0**: <60; **1**: ≥60	kg
V5	Ethnicity	**0**: Arab/others; **1**: European	-
V6	Hypertension history	**0**: Record of hypertension that required medical treatment); **1**: No	-
V7	Kidney function (GFR)	**1**: <30; **2**: ≥30 and <50; **3**: ≥50	mL/min/1.73 m^2^
V8	Previous stroke history	**0**: History of stroke or TIA; **1**: No	-
V9	Previous bleeding history	**0**: History of bleeding; **1**: No	-
V10	Concomitant use of drugs	**0**: Concomitant use of verapamil, diltiazem, antithrombotic agent, NSAID, or COX inhibitor; **1**: No	-
V11	History of MI	**0**: History of MI; **1**: No	-
V12	History of DM	**0**: History of DM; **1**: No	-
V13	History of CHF	**0**: Medical history of CHF or heart echo revealed ejection fraction <40%; **1**: No	-
V14	Smoking	**1**: Never; **2**: Current smoker; **3**: Former smoker	-
V15	History of systemic embolism	**0**: History of systemic embolism; **1**: No	-
V16	Liver function abnormality ^#^	**0**: Presence of liver function abnormality **1**: No	-
V17	Anemia	**0**: Hemoglobin ≥10; **1**: <10	g/dL
V18	Medicine dosage (dabigatran)	**0**: 110 mg twice per day **1**: 150 mg twice per day	-
P1	Vascular events ^†^	**0**: No vascular event happened within the first year of follow-up **1**: Yes	-
P2	Bleeding events *	**0**: No bleeding event happened within the first year of follow-up **1**: Yes	-

Abbr.: BMI, body mass index; GFR, glomerular filtration rate; TIA, transient ischemic attack; NSAID, nonsteroidal anti-inflammatory drug; COX, cyclooxygenase; MI, myocardial infarction; DM, diabetes mellitus; CHF, congestive heart failure. ^#^ Liver function abnormality defined as a medical history of cirrhosis or abnormal biochemical data when the patients were enrolled (bilirubin level more than two times the upper limit of normal, plus one or more of aspartate transaminase, alanine transaminase, or alkaline phosphatase level more than three times the upper limit of normal). ^†^ Vascular events defined as stroke, myocardial infarction, systemic embolism, and vascular death. * Major bleeding was defined as blood loss with a decrease in hemoglobin level of ≥2 g/dL (1.2 mmol/L), transfusion of ≥2 packed red blood cells, or symptomatic bleeding in a critical area or organ. Critical areas were intraocular, intracranial (including hemorrhagic stroke), intraspinal, intramuscular with compartment syndrome, retroperitoneal, intraarticular, or pericardial.

**Table 2:** Subjects’ demographics.

Characteristics	Metrics
V1 Sex	**N (%)**
0: Male	7519 (63.70)
1: Female	4284 (36.30)
V2 Age (years)	**N (%)**
1: <65	1982 (16.79)
2: ≥65 and <75	5123 (43.41)
3: ≥75	4697 (39.80)
V3 BMI (kg/m^2^)	**N (%)**
1: <18.5	123 (1.04)
2: ≥18.5 and <30	7589 (64.30)
3: ≥30	4091 (34.66)
V4 Body weight	**N (%)**
0: <60	1098 (9.30)
1: ≥60	10,705 (90.70)
V5 Ethnicity	**N (%)**
0: Arab/others	3594 (30.45)
1: European	8209 (69.55)
V6 Hypertension history	**N (%)**
0: Record of hypertension that required medical treatment	9301 (78.80)
1: No	2502 (21.20)
V7 Kidney function (GFR)	**N (%)**
1: <30	45 (0.38)
2: ≥30 and <50	2245 (19.02)
3: ≥50	9513 (80.60)
V8 Previous stroke history	**N (%)**
0: Yes	2366 (20.05)
1: No	9437 (79.95)
V9 Previous bleeding history	**N (%)**
0: Yes	774 (6.56)
1: No	11,029 (93.44)
V10 Concomitant use of drugs	**N (%)**
0: Yes	2845 (24.10)
1: No	8958 (75.90)
V11 History of myocardial infarction	**N (%)**
0: Yes	1982 (16.79)
1: No	9821 (83.21)
V12 History of diabetes mellitus	**N (%)**
0: Yes	2739 (23.21)
1: No	9064 (76.79)
V13 History of congestive heart failure	**N (%)**
0: Yes	4125 (34.95)
1: No	7678 (65.05)
V14 Smoking	**N (%)**
1: Never	5781 (48.98)
2: Current	867 (7.35)
3: Former	5155 (43.68)
V15 History of systemic embolism	**N (%)**
0: Yes	306 (2.59)
1: No	11,497 (97.41)
V16 Liver function abnormality	**N (%)**
0: Presence of liver function abnormality	84 (0.71)
1: No	11,719 (99.29)
V17 Anemia	**N (%)**
0: Hemoglobin ≥10 g/dL	11,773 (99.75)
1: Hemoglobin <10 g/dL	30 (0.25)
V18 Medicine dosage (dabigatran)	**N (%)**
1: 110 mg	5870 (49.73)
2: 150 mg	5933 (50.27)
P1 Vascular events	**N (%)**
0: No	11,485 (97.31)
1: Yes	318 (2.69)
P2 Bleeding events	**N (%)**
0: No	9565 (81.04)
1: Yes	2238 (18.96)

Abbr.: BMI, body mass index; GFR, glomerular filtration rate.

**Table 3:** Summary of the values of the hyperparameters which train the best NB, RF, CART, and XGBoost models.

Methods	Hyperparameters	Best Value	Meanings
CART	minispilt	20	The minimum number of observations that must exist in a node for a split to be attempted.
	minibucket	20	The minimum number of observations in any terminal node.
	maxdepth	10	The maximum depth of any node of the final tree.
	xval	10	Number of cross-validations.
	cp	0.0013	Complexity parameter: The minimum improvement in the model needed at each node.
RF	ntree	500	The number of trees in forest.
	mtry	2	The number of predictors sampled for splitting at each node.
NB	fL	1	Adjustment of Laplace smoother.
	usekernel	FALSE	Using kernel density estimate for continuous variable versus a Gaussian density estimate.
	adjust	1	Adjust the bandwidth of the kernel density.
XGBoost	nrounds	100	The number of boosted trees.
	maximum depth	2	The maximum depth of a tree.
	learning rate	0.4	Shrinkage coefficient of tree.
	gamma	0	The minimum loss reduction.
	subsample	1	Subsample ratio of columns when building each tree.
	colsample_bytree	0.8	Subsample ratio of columns when constructing each tree.
	rate_drop	0.01	Rate of trees dropped.
	skip_drop	0.95	Probability of skipping the dropout procedure during a boosting iteration.
	min_child_weight	1	The minimum sum of instance weight.

Abbr: CART, classification and regression tree; RF, random forest; NB, naive Bayes; XGBoost, eXtreme gradient boosting.

**Table 4:** Performance of the four machine learning methods in predicting (a) vascular events and (b) bleeding.

Methods	Accuracy Mean (SD)	Sensitivity Mean (SD)	Specificity Mean (SD)	AUC Mean (SD)
**(a) Vascular events**				
LGR	0.574 (0.03)	0.571 (0.04)	0.707 (0.03)	0.674 (0.00)
NB	0.569 (0.03)	0.565 (0.03)	0.711 (0.04)	0.674 (0.00)
**RF**	**0.890 (0.03)**	**0.898 (0.03)**	**0.599 (0.04)**	**0.780 (0.01)**
CART	0.599 (0.09)	0.598 (0.10)	0.621 (0.09)	0.637 (0.00)
**XGBoost**	**0.646 (0.09)**	**0.645 (0.09)**	**0.693 (0.04)**	**0.717 (0.04)**
**(b) Bleeding**				
LGR	0.604 (0.03)	0.622 (0.05)	0.527 (0.05)	0.605 (0.00)
NB	0.599 (0.01)	0.613 (0.02)	0.537 (0.02)	0.603 (0.00)
**RF**	**0.757 (0.01)**	**0.822 (0.02)**	**0.479 (0.02)**	**0.684 (0.00)**
CART	0.787 (0.07)	0.959 (0.12)	0.052 (0.16)	0.467 (0.03)
**XGBoost**	**0.625 (0.03)**	**0.650 (0.05)**	**0.517 (0.05)**	**0.618 (0.00)**

Abbr.: SD, standard deviation; AUC, area under the receiver operating characteristic curve; LGR, logistic regression; NB, naive Bayes; RF, random forest; CART, classification and regression tree; XGBoost, eXtreme gradient boosting. In predicting both vascular events and bleeding, RF and XGBoost demonstrated higher AUC values (indicated in bold) than LGR.

**Table 5:** Importance ranking of risk factors in predicting vascular events based on RF and XGBoost.

Risk Factors	Average Ranking of 10 Times RF	Average Ranking of 10 Times XGBoost	Average Ranking of the 2 Models	Final Ranking in Predicting Vascular Events
Age	1	5.2	3.1	1
History of congestive heart failure	4.6	2.1	3.35	2
History of myocardial infarction	4	2.8	3.4	3
Smoking	2.2	5.6	3.9	4
Kidney function	5.9	6.1	6	5
BMI	3.5	10.5	7	6
Ethnicity	7.8	7.3	7.55	7
History of diabetes mellitus	8.6	7	7.8	8
Medicine dosage (dabigatran)	8.5	7.5	8	9
Previous stroke history	9.4	9.6	9.5	10
Body weight	12.2	8.9	11.05	11
Concomitant use of drugs	14.3	9.8	12.05	12
Hypertension history	11.8	13.2	12.5	13
Sex	11.7	14.3	13	14
Previous bleeding history	14.5	14	14.25	15
History of systemic embolism	16.3	14.8	15.55	16
Liver function abnormality	16.7	15	15.85	17
Anemia	18	18	18	18

Abbr.: RF, random forest; XGBoost, eXtreme gradient boosting; BMI, body mass index.

**Table 6:** Overall importance ranking of each risk factor in predicting bleeding based on RF and XGBoost.

Risk Factors	Average Ranking of 10 Times RF	Average Ranking of 10 Times XGBoost	Average Ranking of the 2 Models	Final Ranking in Predicting Bleeding
Age	1	1.3	1.15	1
Kidney function	3.2	3.5	3.35	2
Smoking	2.1	4.7	3.4	3
Previous bleeding history	4.7	2.4	3.55	4
Concomitant use of drugs	4.8	5	4.9	5
Medicine dosage (dabigatran)	7	6.7	6.85	6
BMI	5.2	9.6	7.4	7
History of myocardial infarction	9.2	6.1	7.65	8
History of congestive heart failure	10.1	10.3	10.2	9
Ethnicity	9.1	12.2	10.65	10
Sex	10.8	11.3	10.55	11
History of diabetes mellitus	12.8	11.5	12.15	12
Previous stroke history	11	14.2	12.6	13
Hypertension history	14	12.6	13.3	14
Body weight	15	12.3	13.65	15
History of systemic embolism	16	13	14.5	16
Liver function abnormality	17	17	17	17
Anemia	18	17.4	17.7	18

Abbr.: RF, random forest; XGBoost, eXtreme gradient boosting; BMI, body mass index.

**Table 7:** Major nine important variables in predicting vascular events and bleeding.

Average Ranking of Variables	Variable of Prediction of Vascular Events	Variable of Prediction of Bleeding
1	Age	Age
2	History of CHF	Kidney function
3	History of MI	Smoking
4	Smoking	Previous bleeding history
5	Kidney function	Concomitant use of drugs
6	BMI	Medicine dosage (dabigatran)
7	Ethnicity	BMI
8	History of diabetes mellitus	History of MI
9	Medicine dosage (dabigatran)	History of CHF

Abbr.: CHF, congestive heart failure; MI, myocardial infarction; BMI, body mass index.

## Data Availability

The data are available through application to Boehringer-Ingelheim on the research data sharing platform (https://vivli.org/; accessed on 28 July 2021). Restrictions apply to the availability of these data, which were used under license for this study.
